# Identification of Research Priorities in Exercise Oncology: A Consensus Study

**DOI:** 10.7150/jca.42992

**Published:** 2020-02-19

**Authors:** Mhairi Morris, Helen Crank, Mike Loosemore, Clare Stevinson

**Affiliations:** 1 School of Sport, Exercise and Health Sciences, Loughborough University, United Kingdom; 2 National Centre of Sport and Exercise Medicine East Midlands, United Kingdom; 3 Centre for Sport and Exercise Science, Sheffield Hallam University, United Kingdom; 4 National Centre of Sport and Exercise Medicine Sheffield, United Kingdom; 5 Institute of Sport Exercise and Health, University College London, United Kingdom; 6 National Centre of Sport and Exercise Medicine London, United Kingdom

**Keywords:** physical activity, cancer, evidence, consensus

## Abstract

The growth of research in the field of exercise oncology has resulted in a large evidence base for the role of physical activity in preventing and managing cancer outcomes. Nonetheless, there remain many unanswered questions across the multidisciplinary field. This study aimed to determine the priority research questions within exercise oncology using a systematic consensus method. Forty-seven exercise oncology experts engaged in the five-step process of the Nominal Group Technique to generate a list of research questions in small groups and rank the 10 most important. One hundred questions resulted from the process and fifteen received total scores (sum of ranks) of at least 50 from a maximum score of 470. The highest ranked question (score of 125) related to the identification of functional markers of recovery. The next five questions concerned minimum exercise parameters, health professional education, translation of behavioural interventions, effects of exercise on the tumour microenvironment and development of *in vitro* models to study the impact of exercise on cancer cell growth and metastasis. The study has demonstrated the importance of future research across all disciplinary areas of exercise oncology and identified the priority questions to which resources might be directed.

## Introduction

The field of exercise oncology has grown rapidly in recent years and a substantial body of research has accumulated for a role of physical activity in cancer prevention and survivorship outcomes. Most research on risk reduction relies on observational epidemiological studies of total physical activity undertaken across multiple domains (e.g. recreation, occupation, and household, commuting). For research on patient outcomes, experimental studies of structured exercise interventions are used. Studies of physical inactivity as a risk factor have been accruing since 1962 1 resulting in moderate to strong evidence for several common cancers 2,3. Meanwhile, trials of exercise as an intervention for managing treatment side-effects emerged in the 1980s 4,5. The large volume of research generated since these seminal studies has led to evidence-based exercise guidelines for improving aspects of physical function and quality of life 6,7. Alongside these health and wellbeing outcomes, interest has grown in the potential for exercise to influence disease progression and survival. An encouraging number of studies have provided preliminary evidence that physical activity performed after cancer diagnosis reduces risk of recurrence and mortality 3,8.

Despite the considerable progress made in exercise oncology research, it is widely recognised that there are multiple gaps in the current understanding of how to optimise the use of exercise for cancer prevention or recovery 9. Exercise oncology is a multidisciplinary field and research can be broadly categorised into three main areas: 1) cellular and molecular; 2) clinical; and 3) behavioural.

### Cellular and molecular exercise oncology

There are a number of studies into the molecular mechanisms underpinning the impact of exercise on the tumour itself, yet these are still relatively poorly understood (reviewed in 8). Many of these studies are undertaken in animal models of exercise, for example, using voluntary running wheels, forced swimming and forced running on a rodent treadmill, to establish the effects that exercise has on the growth of xenografted human tumours, carcinogen-induced or spontaneously induced tumours in the rodent model 11-13. However, translating these observations from animal model to the *in vivo* human setting is difficult due to interspecies variation, and the effect of a heterologous tumour microenvironment. Fewer studies exist that investigate the impact of human serum extracted following exercise interventions on cancer cell growth *in vitro* 14-16, but as yet, there is no viable *in vitro* system for modelling the effects of exercise on cancer. With the advent of 3D tumour models, bioengineered skeletal muscle tissue that can be electrically and mechanically stimulated to mimic exercise, and microfluidics devices designed to culture multiple tissue types within a controlled environment, there is great potential for developing such *in vitro* systems in the future.

### Clinical exercise oncology

More than 2500 clinical trials have been published providing collective evidence of the benefits of exercise for patient and disease outcomes 6. Several authors have highlighted the limitations of the evidence base in disproportionately focusing on patients with more common cancers 17,18. Additionally, the heterogeneity of exercise interventions in terms of mode, duration and delivery makes it difficult to provide precise conclusions about exercise efficacy for specific outcomes, although recommendations have been possible for some outcomes 6. Recent work in prehabilitation exercise has provided preliminary evidence for faster return of physical function, fewer postoperative complications and shorter hospitalisations 19. To date though there are no data on the impact on treatment success or changes in disease markers.

### Behavioural exercise oncology

The clinical evidence of exercise having positive effects for a range of cancer control outcomes is now substantial. In order to achieve these benefits, individuals must be able to adopt and maintain regular exercise. Behavioural researchers have demonstrated that levels of physical activity are low during and after cancer treatment and remain low in the longer term 20-22. Few reliable predictors of physical activity behaviour in this population have been identified with barriers to participation tending to match those reported in general population samples 23,24. Interventions incorporating behaviour change techniques have been specifically designed to support exercise after a cancer diagnosis. Existing studies have had limited success in changing behaviour 25, and the challenge of exercise promotion remains a strong focus of behavioural research.

### Research priorities

There is general recognition among exercise oncology researchers of the need for greater investigation of this important subject and several authors have suggested future research directions 8,9,26. With there being countless unanswered questions across this multidisciplinary field, it is difficult for researchers and funders to decide where to invest resources. Therefore, this study aimed to determine the priority research questions within across all areas of exercise oncology using a systematic consensus method.

## Methods

### Design

The study was reviewed and approved by Loughborough University Ethics Approvals (Human Participants) Sub-Committee and all participants provided written informed consent. The Nominal Group Technique was adopted to determine the consensus among exercise oncology researchers on the priority questions for the field. The Nominal Group Technique is a structured consensus method that allows all participants an equal opportunity to contribute and employs a quantitative ranking system to establish priority items. This approach has been widely used in health contexts when participants are in a single location and has the advantage of leading to rapid results 27.

### Participants

All 50 delegates of a two-day symposium on exercise oncology were invited to take part in the consensus study and 47 participated. The other three delegates had to leave before the consensus task was completed. Delegates were all academics and/or practitioners working in the field of physical activity and cancer who were invited to attend the symposium due to their expertise on the subject. All participants possessed doctoral degrees (n=21), or medical (n=9) or professional (n=12) qualifications or were working towards doctorates (n=5). They worked at 36 different institutions from Europe and North America with disciplinary backgrounds in molecular biology (n=5), physiology (n=7), medicine (n=5), oncology (n=4), epidemiology (n=3), nursing (n=1), psychology (n=7), dietetics (n=4), and physiotherapy (n=7).

### Procedures

The symposium included invited presentations by three international experts in exercise oncology followed by an open discussion. Study participants were allocated to one of six facilitated groups with seven to eight members for the five-step consensus study. The first step involved outlining to all participants the purpose and procedures of the study. In step two, participants were asked to spend 10 minutes silently thinking of possible questions that should be addressed in future research and write each one individually on sticky note paper. During step three participants took it in turns to read out one question and pass it to the facilitator to place on a flip chart. This process continued until all questions generated had been added to the flip chart. Step four involved the group discussing each question to ensure its meaning was clear and rephrasing if necessary. Any duplicate questions were removed if all group members agreed. The questions from all six groups were then amalgamated and typed as a single list with any repeated questions across groups removed. In the fifth step of the process, participants were given a printed copy of the question list and asked to rank up to ten items for importance (a score of 10 indicated the most important question). Analysis involved summing the sample scores for each question and sorting into descending order. The total score possible for any item was 470 (a maximum score of 10 by all 47 participants).

## Results

**Figure 1:**
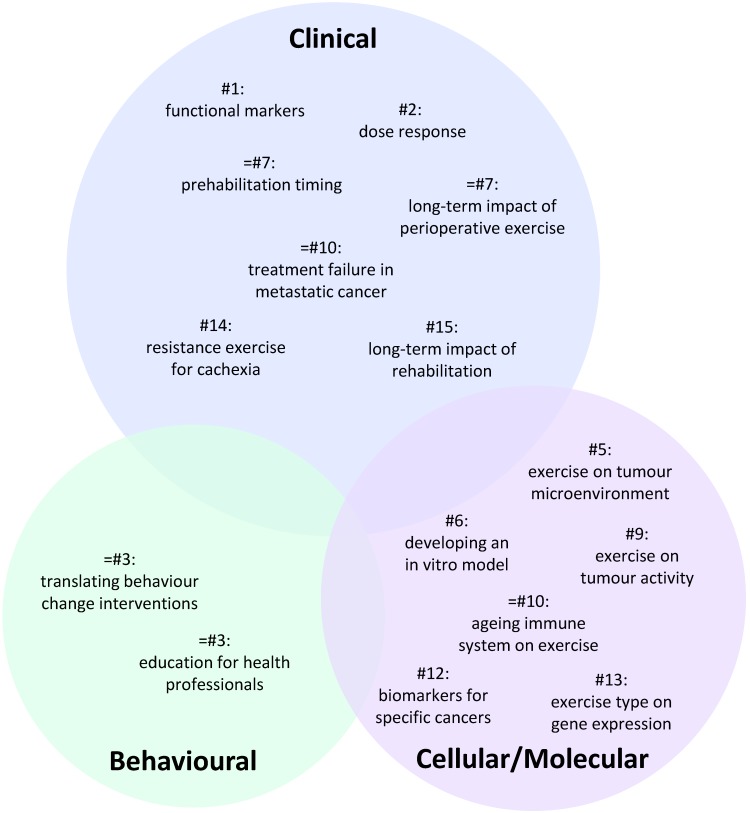
** Venn diagram summarising the distribution of the highest priority research questions across each area of exercise oncology.** Number (#) refers to question ranking. Circle sizes reflect the relative weighting of each the three broad areas. The specific position of individual questions within the circle has no significance.

A total of 100 individual research questions were generated across the six groups Table S1. The 15 highest ranked questions which all received a score of at least 50 are presented in Table 1. A visual representation summarising these questions is presented in Figure 1 reflecting their priority status and cellular/molecular, clinical or behavioural focus.

## Discussion

Although there has been considerable growth in exercise oncology research, there remain multiple gaps in the knowledge base to be addressed. Other authors have proposed key research directions for the field 8,9,26 and this study has used an established systematic process with a large sample of multidisciplinary experts to provide consensus on the current priorities. The results generated important research question across all areas of exercise oncology: clinical, behavioural, and cellular/molecular.

### Clinical exercise oncology research priorities

The highest ranked question related to identifying markers of return to normal function post-therapy. A large body of trials has investigated the effects of exercise interventions on functional outcomes. Significant improvements in cardiorespiratory fitness, upper and lower body strength, and symptoms of fatigue have been observed from aerobic, resistance or combined exercise programs 18. However, whether these or other outcomes can be used to benchmark recovery status is unclear. Preliminary studies indicate that measures of exercise capacity may contribute to prognosis predictions for advanced lung cancers 28,29. Meanwhile, in gerontology there is increasing evidence of grip strength as a marker of frailty 30, brain health 31 and mortality 32. Identifying simple, reliable and valid functional markers that are feasible for oncology practice may help inform personalised care to enhance health, quality of life, and survival outcomes for patients.

The second highest priority question related to minimum exercise parameters. There is limited evidence of the mode, frequency, intensity, or duration of physical activity required for primary prevention or for post-diagnosis outcomes although considerable progress has been made. Three comprehensive reviews of the epidemiological research 2,3,33 suggested that a dose-response relationship was evident for a few cancer sites, but that it is not yet possible to precisely specify the physical activity variables associated with risk reduction. Similarly, an international consensus statement based on an evidence review of cancer survivorship outcomes 6 was able to include exercise prescription guidance for some outcomes (physical function, fatigue, quality of life, mental health). For other outcomes, the authors highlighted the need for continued research to enable greater precision with exercise recommendations. Identifying optimal exercise prescription variable was proposed as a research priority in Courneya et al.'s discussion of the evidence for physical activity and survivorship in 2015 26. The results of the current study indicate that this question remains one of the most important.

### Behavioural exercise oncology priorities

Two questions were ranked equal third highest priority, both relating to behavioural science. One focused on the support and education of healthcare professionals to deliver interventions. Several studies have examined the experiences of oncology staff regarding physical activity promotion among patients. Surveys from the UK 34,35, USA 36, Canada 37 and one with an international sample 38 have demonstrated favourable beliefs among healthcare professionals about the importance of physical activity. The proportion of oncologists who regularly discussed physical activity with patients ranged from 43% 37 to 64% 36. The strongest barriers to discussing this subject with patients were lack of time, expertise, and referral pathways 38. These studies confirm the importance of research to ascertain which healthcare staffs are best positioned to advise patients about physical activity, and what support or education is required.

The other behavioural research question referred to the translation of effective behaviour change interventions into real world settings. Exercise is a challenging behaviour to adopt and maintain and many studies have examined interventions to encourage regular exercise or general physical activity during or after cancer treatment. A systematic review of 27 randomised controlled trials suggested that meaningful increases in exercise could be achieved through a range of intervention approaches 39. These included offering printed or online resources, telephone counselling, and providing supervised exercise sessions. The behaviour change techniques associated with successful findings were social support, graded tasks and action planning. As the evidence base strengthens, it will be important to translate these research-based interventions into community or clinical settings.

### Cellular and molecular exercise oncology priorities

Underpinning the behavioural and clinical research priorities is our understanding of the molecular mechanisms that govern the protective effects of exercise against primary cancer risk, tumour growth and secondary metastases. The next two questions, ranked fifth and sixth, related to the tumour microenvironment and modelling the effects of cancer *in vitro*. Oncologists are becoming increasingly aware of the need to “treat the terrain, not the tumour” yet surprisingly little is known about how exercise modulates the function of cells in distant tissues away from those directly involved in the exercise response 40. In a comprehensive review, Koelwyn et al. (2017) discuss numerous avenues by which exercise might re-programme the tumour microenvironment. The authors note that there is a dearth of research in this area, confirming the importance of developing studies to focus on the effects of exercise on the tumour microenvironment 41.

The second highest priority question in this area, ranked sixth overall, centred around the need for a suitable *in vitro* model for understanding the molecular mechanisms involved in exercise-mediated cancer protection and the harmful effects of adiposity on cancer. Designing an accurate model raises a number of additional questions, including how to accurately recapitulate the tumour microenvironment, how to authentically recreate the effects of exercise, and how to measure the impact of this exercise model on tumour growth and metastasis. Studies in humans are fraught with multiple confounding factors, including the impact of any dietary alterations, age, gender and ethnicity, as well as the genetic and epigenetic differences that accompany these features. The next best alternative - performing studies in mice - has its own disadvantages 42. The paucity of research in this area can, in part, be attributed to the lack of a reliable, repeatable, scalable model for investigation, highlighting the importance of focused efforts to developing such a model.

Whilst *in vitro* models present an excellent low-cost alternative to identifying the molecular events that govern tumour growth and metastasis in response to exercise interventions, conventional 2D monolayer cultures are not representative of the *in vivo* environment. Therefore, there has been a drive towards developing 3D tumour models in recent years 43. Exploiting these advances in 3D cell culture models could lend itself to deepening our understanding of the molecular impact of exercise on cancer cell growth and metastasis, and ultimately aid the design and development of appropriate exercise interventions for specific cancers.

## Limitations

Although this study used an established systematic consensus method to generate data from a large multidisciplinary sample of experts, there are limitations to acknowledge. Firstly, the symposium was conducted at a single site in the United Kingdom, therefore not every invitee was able to attend. Moreover, symposium places were limited to 50 hence the 47 delegates who participated in this consensus process only represented a small proportion of the international pool of expertise.

## Conclusion

This consensus study provides agreement among a large group of experts on the priority questions in the current exercise oncology landscape, and therefore the need for further investigation of this important field. Whilst the need for functional clinical markers was identified as the highest priority research question, the similar rankings between the next five questions demonstrates the near equal importance of each area of exercise oncology research: clinical, behavioural and cellular/molecular. There is a great need to understand the impact of exercise on the tumour itself, the tumour microenvironment, patient prognosis and the impact on chemotherapeutic effectiveness. Progress in these areas will enable the development of exercise prescriptions for specific cancers in different populations that can be effectively translated into practice.

## Supplementary Material

Supplementary table S1.

## Figures and Tables

**Table 1:** Fifteen highest ranked research questions

Rank	Score	Question	Question number
1	125	What markers reflect changes in the ability of patients to return to "normal" function after cancer therapy?	7
2	94	How much physical activity is enough - dose-response intensity and amount?	72
3=	70	What type of support/training/education for health care professionals is required to improve their confidence and competence in delivering physical activity interventions?	24
3=	70	How do we translate effective physical activity behaviour change interventions into real world settings?	30
5	69	What effect does a combined programme of exercise have on the tumour microenvironment?	15
6	64	How do we develop an *in vitro* model for unpicking molecular mechanisms underpinning the protective effects of physical activity on cancer and the harmful effects of adiposity on cancer?	11
7=	63	Does perioperative exercise have a long-term effect on biological markers and cancer recurrence long term?	17
7=	63	What is the optimum timing of exercise pre-chemotherapy to increase efficacy/toxicity?	51
9	59	Does a single bout of exercise modify tumour activity - at a single cell level - in human models of cancer?	13
10=	58	How does the ageing immune system modify the viability of exercise?	14
10=	58	What is the impact of a structured exercise program on time to treatment failure in patients with metastatic/ advanced disease?	60
12	56	Can we develop better biomarkers in the causative pathway for specific cancers for use in clinical trials?	16
13	55	What effect does the type of exercise (e.g. high intensity interval training, strength training) have on gene expression and outcome?	18
14	53	Is resistance exercise useful in combatting cancer cachexia?	64
15	51	What is the long-term impact of participation in a rehabilitation programme for individuals who have completed cancer treatment in terms of cancer recurrence, cancer survival and quality of life?	31
