# Supplementary material for: Identification of Research Priorities in Exercise Oncology: A Consensus Study
Source: J Cancer. 2020 Feb 19;11(9):2702–7. doi: 10.7150/jca.42992 (PMC7066012; doi:10.7150/jca.42992)
Supplement: Supplementary file 1 — Supplementary table S1. [file jcav11p2702s1.pdf]

Table S1. Scores (sum of ranks) for all 100 questions generated by the consensus process

Rank	Total score	Question	Question number
1	125	What markers reflect changes in the ability of patients to return to 'normal' function after cancer therapy?	07
2	94	How much physical activity is enough – dose-response, intensity and amount?	72
3=	70	What type of support/training/education for health care professionals is required to improve their confidence and competence in delivering physical activity interventions?	24
3=	70	How do we translate effective physical activity behaviour change interventions into real world settings?	30
5	69	What effect does a combined programme of exercise have on the tumour microenvironment?	15
6	64	How do we develop an in vitro model for unpicking molecular mechanisms underpinning the protective effects of physical activity on cancer and the harmful effects of adiposity on cancer?	11
7=	63	Does perioperative exercise have a long-term effect on biological markers and cancer recurrence long term?	17
7=	63	What is the optimum timing of exercise pre-chemotherapy to increase efficacy/toxicity?	51
9	59	Does a single bout of exercise modify tumour activity - at a single cell level - in human models of cancer?	13
10=	58	How does the ageing immune system modify the viability of exercise?	14
10=	58	What is the impact of a structured exercise program on time to treatment failure in patients with metastatic/advanced disease?	60
12	56	Can we develop better biomarkers that are in the causative pathway for specific cancers for use in clinical trials?	16
13	55	What effect does the type of exercise (e.g. high intensity interval training~~HIIT~~, strength training) have on gene expression and outcome?	18
14	53	Is resistance exercise useful in combatting cancer cachexia?	64
15	51	What is the long-term impact of participation in a rehabilitation programme for individuals who have completed cancer treatment in terms of cancer recurrence, cancer survival and quality of life?	31
16	47	Do mechanisms vary according to individual physiology/genome?	01
17	43	How can we use longitudinal data from wearable technology/smartphones to predict and individualise interventions to influence activity/lifestyle behaviours?	34
18=	42	What factors contribute to compliance with exercise programmes in cancer patients?	23
18=	42	How do we create personalised physical activity prescription taking into account patient preference?	53
20=	41	Does exercise impact on psychological effects (e.g. neurological changes) to mobilise/activate immune cells?	09

20=	41	What should we measure? Hormones, growth factors, angiogenesis, oxidative mitochondrial damage, telomere shortening?	12
20=	41	What is the optimal time to be active vs rest to achieve disease free survival?	71
23=	37	What aspects of interventions are associated with increased exercise behaviour post 12 months?	22
23=	37	What are the patient perceptions on which health care professionals should deliver physical activity advice/interventions?	26
25	34	What is the long-term role of exercise on the management and treatment of cancer (e.g. tolerability of treatment)	68
26	33	Does exercise work for everyone or can it cause some groups harm?	74
27=	32	Can exercise facilitate oxygenation that enhances immunotherapy and does this enhance survival?	10
27=	32	What exercise programme is best in the following circumstances: a) surgery b) radiotherapy c) chemotherapy?	56
27=	32	Can exercise prevent recurrence?	89
30=	30	Can ~~NHS~~ National Health Service constraints be overcome to enable more exercise and lifestyle interventions within the care pathways?	43
30=	30	How can we use technology (e.g. smartphone applications) to engage young people?	48
30=	30	How can we identify patients that may be at risk of harm from exercise?	92
30=	30	How do identify someone as a responder or non-responder to exercise?	97
34	28	What exercise is actually plausible in a ~~NHS~~ National Health Service setting?	75
35=	27	What are the effects of acute bouts of exercise on blood cell formation in patients with haematological malignancies undergoing chemotherapy/immunotherapy?	05
35=	27	How do we engage hard to reach groups in ~~PA~~ physical activity following cancer diagnosis?	41
37=	26	How can we achieve effective home-based exercise programmes for chemotherapy patients?	19
37=	26	How do we change the behaviour of ~~HCPs~~ healthcare professionals to advocate physical activity to cancer patients?	27
37=	26	What are the contextual issues regarding who the intervention works for, and who it does not work for?	100
40=	25	What are the underlying mechanisms in which exercise (resistance, aerobic or both) help to prevent/treat cancer? E.g. AMP-activated protein kinase~~AMPK~~, hypoxia-inducible factor~~HIF~~, NF-κB (nuclear factor kappa-light-chain-enhancer of activated B cells)~~NFKB~~, inflammation, metabolic, glycolysis, angiogenesis.	06
40=	25	What access to fitness/exercise facilities are provided in hospitals/treatment centres for patients to exercise during hospital admissions and/or outpatient visits?	21
40=	25	Are there synergetic effects of exercise and energy restriction/fasting on chemotherapy toxicity?	84
43=	24	What do cancer survivors understand about exercise and physical activity?	29
43=	24	What is the most effective way to support people to be physically active throughout the cancer journey?	63
43=	24	Can exercise improve/extend overall survival in metastatic disease?	90

46	23	Does ~~PA~~ physical activity as part of a treatment plan in paediatric population improve quality of life and/or treatment outcome?	76
47=	22	Which cancer diagnoses are most likely to benefit from lifestyle interventions?	44
47=	22	What impact do lifestyle interventions have on body composition following cancer diagnosis?	96
49=	21	What are the most appropriate ways to monitor how well patients complete exercise interventions?	33
49=	21	Are some types of exercise best for some cancers?	65
50	20	What is the effect of exercise on secondary cancer?	87
51=	19	Is the optimal dose of exercise dependent on certain factors, such as age or fitness level at diagnosis?	59
51=	19	Does intervening at diagnosis have long term benefits?	82
51=	19	Is there variation in individual response to physical activity?	85
51=	19	Does pre-operative exercise increase post-operative outcomes? If so, how?	95
55=	18	What is the role of ketosis in cancer and exercise?	04
55=	18	How do we educate patients on the importance of exercise and diet for recovery, side effects and recurrence?	45
55=	18	How can we improve clinical confidence in exercise research?	50
58=	17	Does exercise need to be supervised and intensity-dependent to be effective?	80
58=	17	Can a prehabilitation programme of exercise increase the success/tolerability of novel cell-based therapeutics?	98
58=	17	Is exercise training during neoadjuvant cancer treatment safe (frequency, intensity, time, type)?~~(FITT)?~~	99
61=	16	What are the barriers to exercise and how do we use behaviour change models to increase exercise adherence?	46
61=	16	How do we promote physical activity in patients with a history of being sedentary and at risk for developing cancer?	49
61=	16	When is the most beneficial time of day to exercise for different subgroups of patients?	78
64=	15	How does physical activity prevent breast/colorectal cancer? Are the effects different to just energy restriction?	03
64=	15	What is the evidence that cancer biomarkers are directly related to cancer incidence?	08
64=	15	In unfit patients with poor performance status, can exercise improve their performance status so that they are fit enough for treatment?	58
64=	15	What is the role of exercise of body composition, and how should we measure it in ~~NHS~~ National Health Service settings?	86
68	14	How can we get patients exercising in hospital, such as on wards, in chemotherapy units and in clinics?	62
69=	13	What are the barriers to researching the effects of exercise on children and teenagers with cancer?	42
69=	13	How does moderate-vigorous exercise interact with different chemotherapy drugs?	67
71=	12	Do outcomes differ with group training versus one to one?	61

71=	12	What is the safe level of exercise intensity in young people during treatment?	69
71=	12	Can exercise at high intensity cause patients harm during chemotherapy treatment?	91
71=	12	Can ~~PA~~ physical activity overcome adverse effects of sedentary behaviours relevant to cancer?	93
75=	10	What are the physiological effects of sedentary behaviour above what we already know?	02
75=	10	What are the patient belief and barriers to exercise?	38
77=	9	What is the role of health care professionals in exercise in the cancer care pathway?	25
77=	9	How do we increase patient adherence to exercise and lifestyle interventions?	36
77=	9	What are the current ~~PA~~physical activity levels in paediatric patients following cancer diagnosis?	77
80=	8	What is patient preference for location of exercise?	20
80=	8	Who should deliver the exercise programmes for children and young adults and what type of programme is acceptable?	83
82	7	Is there an optimal level of fitness before surgery should be considered?	57
83=	6	What level of intervention is required to increase/ change oncologists' perceptions of patients 'fitness' for chemotherapy/anti-cancer treatment?	32
83=	6	How do we spread the idea of exercise interventions to wider society to help combat barriers faced by patients?	39
83=	6	Are the physical activity guidelines appropriate for patients with cancer and post-cancer?	55
83=	6	When is it optimal to initiate an exercise programme following the diagnosis of cancer?	79
87=	5	How important are the psychosocial impacts of exercise?	47
87=	5	Do exercise programmes increase patient access to surgery?	94
89	4	How can we encourage more ~~HCP~~healthcare professionals to promote ~~PA~~ physical activity in clinical practice? In what setting is most effective, primary or secondary?	73
90=	3	What influences physical activity behaviours?	37
90=	3	Who is best placed to prescribe exercise and how will it be progressed (including the more complex patients)?	54
92	2	Does laboratory-based interval training translate to training in real world and improve quality of life/prevent recurrence?	81
93=	1	When should we educate patients on exercise - post diagnosis, during treatment or post treatment?	40
93=	1	When would patients be most responsive to advice/intervention?	66
95=	0	What advice do ~~HCP~~healthcare professionals give to patients in regard to exercise?	28
95=	0	Does premorbid perception of exercise impact on outcome or is there a teachable moment?	35
95=	0	Are the concepts of increasing physical activity and increasing or starting exercise met differently?	52
95=	0	Regardless of fitness, activity levels and socioeconomical status, are the outcomes of exercise similar?	70

95=	0	Should any extra measures be taken to help enable PICC (peripherally inserted central catheter) lines? Will using swimming be problematic with immune function?	88
